# Does physiotherapy reduce the incidence of postoperative complications in patients following pulmonary resection via thoracotomy? a protocol for a randomised controlled trial

**DOI:** 10.1186/1749-8090-3-48

**Published:** 2008-07-18

**Authors:** Julie C Reeve, Kristine Nicol, Kathy Stiller, Kathryn M McPherson, Linda Denehy

**Affiliations:** 1 Division of Rehabilitation and Occupation Studies, Faculty of Health and Environmental Studies, AUT University, Auckland, New Zealand; 2 Allied Health, Auckland City Hospital, Auckland, New Zealand; 3 Physiotherapy, Royal Adelaide Hospital, Adelaide, South Australia, Australia; 4 School of Physiotherapy, Faculty of Medicine, Dentistry and Health, University of Melbourne, Melbourne, Victoria, Australia

## Abstract

**Background:**

Postoperative pulmonary and shoulder complications are important causes of postoperative morbidity following thoracotomy. While physiotherapy aims to prevent or minimise these complications, currently there are no randomised controlled trials to support or refute effectiveness of physiotherapy in this setting.

**Methods/Design:**

This single blind randomised controlled trial aims to recruit 184 patients following lung resection via open thoracotomy. All subjects will receive a preoperative physiotherapy information booklet and following surgery will be randomly allocated to a Treatment Group receiving postoperative physiotherapy or a Control Group receiving standard care nursing and medical interventions but no physiotherapy. The Treatment Group will receive a standardised daily physiotherapy programme to prevent respiratory and musculoskeletal complications. On discharge Treatment Group subjects will receive an exercise programme and exercise diary to complete. The primary outcome measure is the incidence of postoperative pulmonary complications, which will be determined on a daily basis whilst the patient is in hospital by a blinded assessor. Secondary outcome measures are the length of postoperative hospital stay, severity of pain, shoulder function as measured by the self-reported shoulder pain and disability index, and quality of life measured by the Medical Outcomes Study Short Form 36 v2 New Zealand standard version. Pain, shoulder function and quality of life will be measured at baseline, on discharge from hospital, one month and three months postoperatively. Additionally a subgroup of subjects will have measurement of shoulder range of movement and muscle strength by a blinded assessor.

**Discussion:**

Results from this study will contribute to the increasing volume of evidence regarding the effectiveness of physiotherapy following major surgery and will guide physiotherapists in their interventions for patients following thoracotomy.

**Trial registration:**

The study protocol is registered with the Australian and New Zealand Clinical Trials registry (ANZCTRN12605000201673).

## Background

Physiotherapy interventions have been regularly utilised in the prevention and treatment of both pulmonary and musculoskeletal complications following major surgery since the 1960s. Following thoracotomy, postoperative pulmonary complications (PPCs) are an important cause of morbidity, contributing to significant increases in health care costs, length of intensive care and hospital stay and patient discomfort [1-5]. In some major surgical patient groups there has been a steadily accumulating body of evidence demonstrating that postoperative *prophylactic *physiotherapy for the prevention of PPCs and musculoskeletal problems may be unnecessary [6-12]. Whilst physiotherapy for thoracic surgical patients continues to be strongly advocated, to date there have been no randomised controlled trials supporting its effectiveness. Evidence from a recent cross sectional study with historical controls in patients following lung resection suggests that physiotherapy may reduce length of hospital stay and incidence of atelectasis (with a subsequent reduction in hospital costs) but appears to have no influence over the incidence of pneumonia and overall morbidity [13]. Other research has demonstrated that adding various adjuncts (such as incentive spirometry) to usual care physiotherapy treatment programmes makes no significant difference to outcome, however to date there have been no randomised controlled trials including a no treatment group [14-17]. It remains unknown whether prophylactic respiratory physiotherapy, as part of inpatient postoperative recovery, is necessary following pulmonary resection and clarification of the role and efficacy of these interventions is overdue.

Thoracotomy may also lead to chronic pain and long term restriction of shoulder function and physiotherapy and early mobilisation are commonly recommended and implemented to prevent these problems [18,19]. Whilst shoulder range of movement, strength and function has been extensively examined in this patient group [20-24] the role of a postoperative physiotherapy exercise programme in minimising this dysfunction has not been investigated. Significant correlation between upper limb morbidity and poorer quality of life has been reported in patients in other surgical groups [25]. A study to evaluate the effectiveness of a shoulder and thoracic cage exercise programme following thoracotomy should further develop an understanding of both short and long term shoulder morbidity in this patient group.

The Physiotherapy Management of the patient undergoing Thoracic Surgery study (PMoTS) was designed as there are no published RCTs which address these outcomes.

### Aims and Hypotheses

Thus the primary aims of the study are to:

1. Compare the incidence of PPCs between groups

2. Compare the postoperative length of hospital stay (LOS) between groups

The secondary aims are to:

3. Compare LOS in those subjects who develop PPCs and those who do not.

4. Compare the recovery of shoulder function, shoulder range of motion (ROM), and shoulder muscle strength (MMS) between groups

5. Compare the health related quality of life (HRQoL) between groups

The study hypothesis for the primary aims is:

H1. Routine postoperative prophylactic physiotherapy will significantly reduce the incidence of PPCs and LOS compared to no postoperative physiotherapy following open thoracotomy.

The hypothesis for the secondary aim is:

H2. A postoperative exercise programme will significantly improve the recovery of shoulder ROM, muscle strength and function following open thoracotomy.

## Methods and Design

### Study design

The PMoTS study is a single blinded randomised controlled trial of patients undergoing elective pulmonary resection via open thoracotomy in one thoracic surgical unit in New Zealand. The study aims to investigate the effectiveness of physiotherapy treatments following surgical removal of part or whole of one lung.

### Subjects

#### Inclusion/exclusion criteria

To be eligible for enrolment subjects must be adults admitted to Auckland City Hospital, New Zealand to undergo elective lung resection via open thoracotomy. They must understand written and spoken English and give informed written consent. Subjects will be excluded if they are unable or unwilling to comply with treatment, have tumour invasion into the chest wall or brachial plexus, present with a PPC prior to randomisation on postoperative day one, remain ventilated for longer than 24 hours following surgery or have received physiotherapy for shoulder problems or respiratory problems within two weeks prior to admission for surgery. Subjects with postoperative neurological or mobility complications requiring comprehensive physiotherapy input to progress towards discharge (defined as being more than two physiotherapy interventions), will be provided with the physiotherapy as required, remain in the group to which they were allocated and analysed using intention to treat principles. Subjects, who live within a 60 km radius of Auckland Hospital and can attend outpatient appointments, will form a subgroup that, in addition to all other measures, will attend for follow up shoulder ROM and muscle strength measures.

#### Sample size

Sample size was calculated for the primary outcome measure of PPC with a type I error rate of .05 and a type II error rate of 0.20 (80% power). Estimates of the incidence of PPCs vary throughout the literature dependent upon criteria used for diagnosis but best estimates suggest a PPC rate (after thoracotomy and amenable to physiotherapy interventions) of between 10–25% occurs in this population with routine physiotherapy care [4,14], Assuming a PPC rate of 20% in the Control Group and 5% in the Treatment Group (thus a 15% difference in PPCs), 84 subjects in each group are required. Allowing for an estimated loss to follow up rate of 9% (based on similar studies) a sample size of 184 subjects (92 per group) is necessary to demonstrate sufficient power for the primary outcome. Because there are no randomised trials that include a control group on which to accurately base this sample size calculation, an interim analysis will be undertaken when total n = 80.

### Ethics and study registration

**Figure 1:**
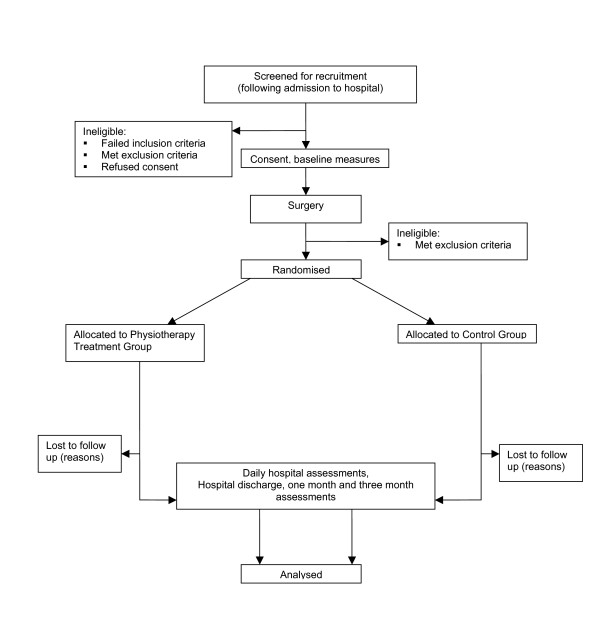
Trial protocol.

Ethical permission for the study was granted from Northern X Regional Ethics Committee in October 2005. The study protocol is registered with the Australian and New Zealand clinical trials registry (ANZCTRN12605000201673). The flow of subjects through the study follows the CONSORT (Consolidated Standards of Reporting Trials) recommendations (see Figure 1).

### Procedures

#### Recruitment and randomisation

Subjects eligible to take part are screened from the thoracic surgical operating list by the study investigators (JR & KN) on admission to hospital. If eligible to take part the study investigators approach the subject to explain the study and gain informed consent. It is explained that they may withdraw from the study at any time without jeopardising their future care. On day one postoperatively, prior to any physiotherapy interventions and following extubation, subjects are given a consecutive study number and randomly allocated to the Treatment Group or Control Group by an independent physiotherapist. The ward physiotherapist is then told of the group allocation which is recorded and the envelope stored. Group allocation is assigned using a computer generated random allocation (from ) and individual group assignment is kept in sealed opaque envelopes.

In the event of more than one thoracic surgical procedure per day randomisation is performed according to order of admission onto the postoperative intensive care/surgical ward. To avoid influencing postoperative regimens, subjects taking part in the research trial are, where possible, allocated to separate rooms during their hospital stay.

In the subgroup who meet the inclusion criteria for shoulder ROM and muscle testing, a computer generated randomised order for performing the ROM and muscle strength measures is determined before preoperative measures and adhered to throughout the study. The randomisation order is generated from .

### Interventions

#### a. Preoperative education

After consent all subjects have preoperative demographic data recorded in order to compare baseline measures between groups (see Table 1), and are given a physiotherapy written information sheet by a study investigator. This broadly explains the need for postoperative breathing and coughing exercises, early ambulation, and shoulder mobility exercises. It outlines breathing exercises, huffing/coughing and the importance and progression of ambulation postoperatively. It gives general advice about shoulder and arm use in the postoperative period. No other form of preoperative physiotherapy education and treatment is provided to subjects in either group.

**Table 1:** Demographic Data

▪ Age
▪ Ethnicity
▪ Sex
▪ Smoking history & pack year history
▪ Body mass index (BMI)
▪ American Society of Anaesthesiologists score (ASA)
▪ Hand dominance
▪ History and symptoms of chronic lung disease
▪ Relevant past medical history
▪ Percutaneous oxygen saturation (SpO_2_)
▪ Pulmonary function tests
◦ Forced Expiratory Volume in 1 second (FEV1)
◦ Forced Vital Capacity (FVC)
◦ FEV1/FVC
▪ Date of surgery
▪ Surgeon
▪ Surgical procedure
▪ Duration of anaesthesia
▪ Incision site/type of thoracotomy (muscle sparing, postero-lateral, antero-lateral, axillary)
▪ Rib resections
▪ Number of chest drains in situ, length of time on suction (number of days) and length of time in situ (number of days) postoperatively
▪ Postoperative analgesia and method of administration
▪ Relevant past medical history of shoulder or upper back/neck problems and management
▪ Presence of seroma at wound site (Y/N)
▪ Time to first sit out of bed (number of hours postoperatively)
▪ Time to first mobilisation > 10 metres or equivalent walk on spot if chest drains remain on suction (number of days postoperatively)
▪ Reason for increased length of stay
▪ Physiotherapy interventions administered (total number of sessions, cumulative time and type of intervention)
▪ Return to the intensive care unit and operating theatre
▪ Postoperative chemotherapy or radiotherapy

#### b. Postoperative treatment

Both Treatment and Control Group subjects will receive standard nursing and medical care according to the clinical pathway in use at the unit where the research is being undertaken.

**Figure 2:**
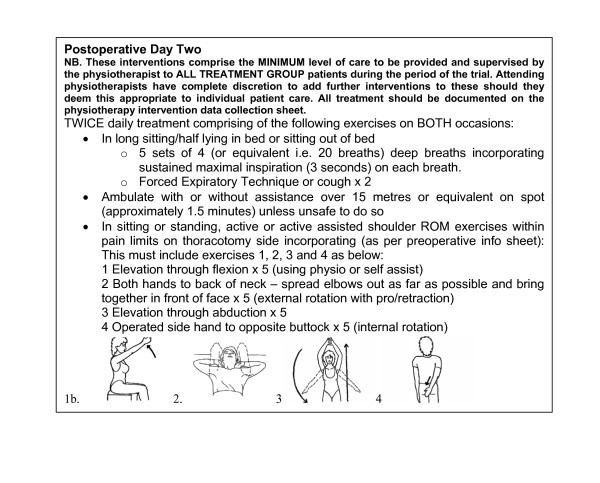
Example of minimum physiotherapy intervention programme.

Subjects in the Treatment Group will receive postoperative physiotherapy from the attending ward physiotherapist as per a written protocol on a daily basis until discharge. This includes a *minimum *predetermined amount of: deep breathing and coughing exercises, assistance with ambulation, and a progressive shoulder and thoracic cage mobility exercise programme (see Figure 2). Subjects will be encouraged by the attending physiotherapist to practice the exercises outside physiotherapy treatment times but this practice will not be quantitatively measured. Upon discharge from hospital Treatment Group subjects will receive a discharge exercise and advice sheet with an exercise diary explained by the attending ward physiotherapist.

Control Group subjects will receive no postoperative physiotherapy. They may be verbally encouraged by attending nursing staff to practice the exercises outlined on their preoperative physiotherapy information sheet throughout their postoperative stay.

### Outcome measurement

Outcome measures, instruments used and timeframes for measurement are summarised in Table 2.

**Table 2:** Summary of outcome measurements

**Primary Outcomes**	**Measurement instrument**	**Time point**
PPC	8 point item score (see Table 3)	Postop daily to discharge

**Secondary Outcomes**	**Measurement instrument**	**Time point**

LOS	Retrospective from patient information database	At discharge
SPADI	13 items scored on 11 point Likert scale	Preop, at discharge, 1 month postop, 3 months postop
ROM of shoulder flexion, elevation through abduction and external rotation (subgroup)	Plurimeter – V inclinometer	Preop, at discharge, 1 month postop, 3 months postop
MMS of shoulder abduction, flexion and internal rotation (subgroup)	Hand held dynamometer (Lafayette Instruments)	Preop, 1 month postop, 3 months postop
HRQoL	Short Form 36 v2 (NZ)	Preoperative, 1 month postop, 3 months postop
Pain	Body charts and verbal rating scale (end descriptors 0 = no pain and 10 = worst pain possible)	Preop, at discharge, 1 month postop, 3 months postop

Key : HRQoL – Health related quality of life, LOS – length of postoperative hospital stay, MMS – muscle strength, NZ – New Zealand, Postop – postoperative, PPC – postoperative pulmonary complication, Preop – preoperative, ROM – range of movement, SPADI – shoulder pain and disability index.

#### 1. PPC

Will be diagnosed on a daily basis until hospital discharge using the diagnostic tool shown in Table 3. The data will be collected by an attending physiotherapist and phoned to a blinded assessor who determines the diagnosis of a PPC.

**Table 3:** Postoperative pulmonary complication diagnostic tool

**During this trial the following criteria will be used to diagnose PPCs. Patients must be assessed before 11 am daily.**

**PPCs**
For the purposes of this study PPC will be diagnosed by presence of 4 or more of the following:
1. Chest radiograph report of atelectasis/consolidation. In the event of no CXR being taken, the CXR report from the previous postoperative day will be used. If neither are available a not available will be reported (n/a). If a CXR report is not available but a CXR has been taken a ward medical officer will be asked to report on this should this be the defining criteria for PPC.
2. An otherwise unexplained WCC of >11.2 × 10^9^/L *or *administration of respiratory antibiotics postoperatively (in addition to those administered routinely postoperatively). In the event of no WCC being taken, the WCC report from the previous postoperative day will be used. If none of these are available a n/a will be reported
3. Fever as seen by raised oral temperature >38°C with no focus outside of the lungs. The highest temperature within the previous 24 hours will be reported.
4. Positive signs of infection on sputum microbiology.
5. Production of purulent (yellow or green) sputum differing from preoperative status
6. SpO_2 _< 90% on room air (see measurement protocol below).
7. Diagnosis of pneumonia/chest infection by attending physician.
8. Re-admission to the ITU/HDU with problems which are respiratory in origin or a prolonged stay on the ITU/HDU (over 36 hours) with problems which are respiratory in origin.

***Sp0_***2 ***_measurement***
All SpO_2 _measurements will be taken in the morning prior to physiotherapy treatment. Prior to measurement of SpO_2_:
▪ The patient will be positioned in upright sitting (or long sitting if unable to be out of bed).
▪ O_2 _therapy will be withdrawn for a period of 5 minutes & SpO_2 _will be monitored but not recorded during this time. NB if patient on room air allow monitor to stabilise for 1 minute prior to reading.
▪ Measurement will be by designated pulse oximeter via a finger probe.
▪ After 5 minutes the SpO_2 _will be measured by reapplying the finger sensor to the index finger of one hand for 30 seconds.
▪ The lowest SpO_2 _during the 30 second measuring period will be recorded.
▪ If a patient's SpO_2 _drops below 88% at any stage of the measures they will be immediately returned to supplemental O_2 _as prescribed and measures abandoned. This will be noted and the value recorded.
▪ If the SpO_2 _drops below 90% (i.e. 89% or below) this will be noted as achieving as one of the criteria for PPC.
▪ Only patients with an SpO_2 _of 89% or below will not be taken off oxygen for SpO_2 _monitoring purposes (i.e. these patients will have already achieved criteria for PPC without removal from O_2_).
▪ If the physiotherapists notes the hands to be cool, peripheral shutdown, poor pulsatile flow on the SpO_2 _monitor or a dampened trace this will be recorded as being unreliable (N). A reliable trace will be recorded as (Y).

Key: CXR – Chest X Ray, SpO_2 _– Percutaneous oxygen saturation, PPC – postoperative pulmonary complication, WCC – white cell count, HDU – high dependency unit, ICU – intensive care unit.

#### 2. Length of Stay (LOS)

Length (number of days) of postoperative stay will be measured from data held on the hospital database.

#### 3. Shoulder function

Will be measured using the Shoulder Pain and Disability Index (SPADI) preoperatively, on discharge from hospital, one month and three months postoperatively. This is a self reported shoulder specific index developed by Roach et al. (1991) [26] which consists of 13 items in two subscales: pain (5 items) and disability (8 items). Each item is measured on an 11 point Likert scale where 0 = "no pain/no difficulty" and 11 = "worst imaginable pain/so difficult it required help" on each subscale respectively. The SPADI is scored by adding then averaging the two subscales to determine a score out of 100. A higher score means greater pain/disability. The SPADI has been demonstrated to detect change in subject's status over time, have good test-retest reliability, internal consistency and good criterion and construct validity although not within the thoracic surgical population [26-28].

#### 4. Shoulder ROM

In a subgroup of patients living within a 60 km radius of Auckland Hospital, active ROM will be measured preoperatively, on discharge from hospital, one month and three months postoperatively by a blinded assessor using digital inclinometry via a Plurimeter-V inclinometer. ROM will be measured in those muscle groups which are divided during surgery thus potentially affecting range of movement. The measures are:

a. Total shoulder flexion (the movement includes scapular motion)

b. Total shoulder elevation through abduction (the movement includes scapular motion)

c. Glenohumeral external rotation

All ROM measures (except external rotation) are taken with the subject fully upright in a standardised chair with the opposite forearm resting on a fixed table to eliminate trunk movement. Sitting has been chosen as the starting position for most measures due to the possibility of subjects with respiratory disorders being unable to tolerate supine lying. The position of the inclinometer is established by measurement from a clear surface marking (e.g. 1 cm above lateral epicondyle).

▪ Shoulder flexion is measured with the arm at the side, elbow extended, shoulder in neutral rotation with palm facing the thigh and thumb facing forwards. The subject actively flexes the arm with the thumb leading throughout, maintaining elbow extension.

▪ Shoulder elevation is measured with the arm at the side, elbow extended, arm in external rotation with the palm facing forward. The subject actively elevates the arm leading with the thumb maintaining elbow extension.

▪ Shoulder external rotation is measured in supine lying (on a standardised bed) with the arm at side, humerus supported by the bed, elbow flexed to 90°, forearm in the mid prone position, hand in fisted position, pointing towards the ceiling. The subject actively externally rotates the arm maintaining the elbow on the bed and leading with the dorsum of the hand.

For all movements care is taken to avoid trunk assisted movements. On each occasion the arm is returned to 0° and each movement is repeated twice after one practice measure. These measures, where possible, follow a standardised protocol that has been shown to have acceptable intra and interrater reliability for use within a randomised controlled trial [29]. As well as recording ROM subjects report maximum pain during each movement on a 0 – 10 verbal rating scale. The maximum reading for pain for each movement is recorded.

#### 5. Isometric muscle strength

In a subgroup of patients living within a 60 km radius of Auckland Hospital, muscle strength will be measured preoperatively, one month and three months postoperatively by a blinded assessor using a handheld manual muscle tester (Lafayette instruments) which has been shown to have moderate to high interrater and intrarater reliability [30]. Measurements will be made of those of muscle groups that have been divided during surgery and thus may affect muscle strength. The measures are:

a. Shoulder flexion

b. Shoulder abduction

c. Shoulder internal rotation

d. Shoulder extension

All measures are taken in the same position as described for ROM testing with the position of the dynamometer established in the same way using the easily identifiable surface markings of 1 cm above the elbow crease (for flexion), 1 cm above the lateral epicondyle (for abduction), 1 cm above the olecranon (for extension) or the skin crease (usually middle) between the radial styloid process and the head of the ulna (for internal rotation). The starting position is with the arm at the side with one gripped fist width between the distal end of the humerus and chest wall, the elbow flexed to 90° and the forearm in the mid prone position. This position was chosen due to the potential for subjects to be unable to commence muscle strength measures in 90° or 45° glenohumeral abduction as recommended by some authors [31,32]. Resistance is applied against the direction of shoulder movement for three to five seconds. The "make" (rather than "break") technique is used requiring the examiner to resist a maximal voluntary contraction by the subject which is essentially an isometric contraction and has been shown to have high interrater and intrarater reliability even in inexperienced examiners [33].

Standardised instructions and verbal encouragement is given and, after one practice contraction, each movement is measured three times with one minute between measures. Subjects report maximum pain during each movement on a 0 – 10 verbal rating scale. In keeping with other authors the maximum intensity of pain for each movement will be recorded [33-35].

#### Demographic and background information

Table 1 gives subject and operative data that will be collected and used to compare characteristics that could influence the outcome of the study. These data will be collected by the study investigators from subjects' medical records. In addition, records of any postoperative radiotherapy, chemotherapy and information regarding any additional shoulder treatments from health care professionals will be sought.

### Data analysis

Analyses will be conducted using an intention to treat principle for all randomised subjects. Demographic data will be analysed using descriptive statistics, Chi squared and Independent t tests or parametric equivalents.

A Chi squared test will be used to determine if there is any significant difference in the incidence of PPC between groups. LOS will be tested for normality of data. If normally distributed an Independent t test will be used to calculate the differences in mean LOS between groups and in differences in LOS in those subjects who do and do not develop PPCs. Data will be presented as mean differences with 95% confidence intervals. If data are not normally distributed a non parametric Mann Whitney U test will be used. The absolute risk reduction and number needed to treat will also be calculated for the primary outcome of PPC. Further between group comparisons for repeated measures of interval data (such as shoulder ROM, shoulder muscle strength, ADL and HRQoL) will be made using repeat measures ANOVA. Significant results will be analysed using post hoc tests such as the Scheffe F test. The alpha level for all statistical analyses will be set at 0.05.

## Discussion

This study uses a single blind randomised controlled design to investigate the efficacy of physiotherapy interventions in the prevention of complications following pulmonary resection via open thoracotomy. The benefits of physiotherapy for these patients have not been established despite being widely recommended as an integral part of standardised postoperative care. To our knowledge this will be the first randomised controlled trial using a Control Group with no physiotherapy to study the efficacy of physiotherapy interventions in preventing pulmonary and shoulder complications in this population.

The role of prophylactic physiotherapy, particularly the value of deep breathing exercises, following other types of major surgery has been recently challenged as a result of evidence from RCTs [36,37]. Prior to the current study being initiated, we determined, using a postal survey, that the majority of physiotherapists continue to prophylactically treat patients after open thoracotomy [19]. Most commonly deep breathing and airway clearance manoeuvres, early ambulation and shoulder exercises were used for patients following thoracic surgery [19]. Whether this level of intervention is necessary in this patient group is currently unknown. Given the findings of the survey which determined commonality in practices across physiotherapy providers, we have chosen to use standardised minimum physiotherapy interventions (see example in Figure 2) rather than utilising a pragmatic design where therapists would be free to choose their own interventions. This will enable both reproducibility and consistency. Delivery of the programme is by the attending ward physiotherapist whose identity will vary according to workload allocations thus increasing the external validity of the study. Deviations from the standardised protocol will be recorded.

For all subjects, the self reported measures of shoulder function will provide clinically relevant measures of shoulder recovery following surgery. Studies have shown ROM impairment and functional status to be significantly associated [38,39] and therefore any subject not achieving recovery of shoulder function scores within 20 per cent of preoperative status will be offered outpatient follow up physiotherapy as necessary. As subjects undergoing pulmonary resection in our surgical unit are drawn from a wide geographical area, follow up measures of shoulder ROM and MMS are not feasible for all patients. Thus we determined that patients living within 60 kilometres of the unit who were able to attend outpatient appointments will form a subgroup who will undergo ROM and muscle strength testing as additional measures.

It is anticipated that data collection will be completed by late 2008 and that this study will contribute to the increasing evidence base into the effects of physiotherapy interventions following major surgery.

## Competing interests

The authors declare that they have no competing interests.

## Authors' contributions

JR, LD, KS and KM designed the trial protocol. JR, KN, LD, and KS procured the study funding. KN is the project manager. JR drafted the manuscript and LD, KS and KM contributed to the manuscript. All authors read and approved the final manuscript.
